# Pleomorphic Adenoma of the Cervical Heterotopic Salivary Gland: A Case Report

**DOI:** 10.1155/2012/470652

**Published:** 2012-02-07

**Authors:** Saman Vegari, Masoud Naderpour, Alireza Hemmati, Hosein Baybordi

**Affiliations:** ^1^Department of Otolaryngology-Head and Neck Surgery, Tabriz University of Medical Sciences, Tabriz 5174867773, Iran; ^2^Department of Pathology, Tabriz University of Medical Sciences, Tabriz 5174867773, Iran

## Abstract

*Introduction*. Although pleomorphic adenoma is the most common neoplasm of the salivary glands, this tumor most commonly involves the minor salivary glands of palatal and rarely occurs in cervical region. *Case Report*. A 21-year-old female referred to our clinic due to painless mass of right upper region of neck. After paraclinical and pathologic evaluation, it was diagnosed as cervical pleomorphic adenoma. *Conclusion*. Pleomorphic adenoma may be rarely involving the neck. Although the prognosis is good, the choice treatment is the complete resection of the tumor.

## 1. Introduction

 There are nearly 450 to 750 minor salivary glands throughout the upper aerodigestive tract [1]. Aberrant salivary glands tissue may occur in a variety of locations such as lymph nodes, especially in parotid area. It is also reported in one percent of tonsillar tissue. Other reported sites are mandible, lower neck, hypopharynx, middle ear, sternoclavicular joint, thyroglossal duct, and pituitary gland. When these tissues become neoplastic, the type of tumor may be mucoepidermoid carcinoma, adenocystic carcinoma, or adenocarcinoma, in a descending order of frequency [2].

One type of these neoplastic lesions most commonly involving the salivary glands is Pleomorphic adenoma. This tumor also has been called benign mixed tumor. Pleomorphic adenoma constitutes about 75% of all benign tumors of the major salivary glands. This tumor most commonly occurs in the minor salivary glands of palatal area [1–3].

## 2. Case Report

A 21-year-old female patient was referred to our clinic in Imam Reza hospital with painless mass of right upper part of her neck from seven years ago. No significant changes had developed in its size during this period. Physical examination revealed a mass in the upper border of thyroid cartilage next to bifurcation of common carotid. The mass appeared with no tenderness. It was also mobile, firm, and nonpulsatile. There was no bruit in auscultation. The examination of the head and neck including nasopharynx, oral cavity, hypopharynx, and larynx was normal. All of the routine blood chemistry tests were normal.

**Figure 1:**
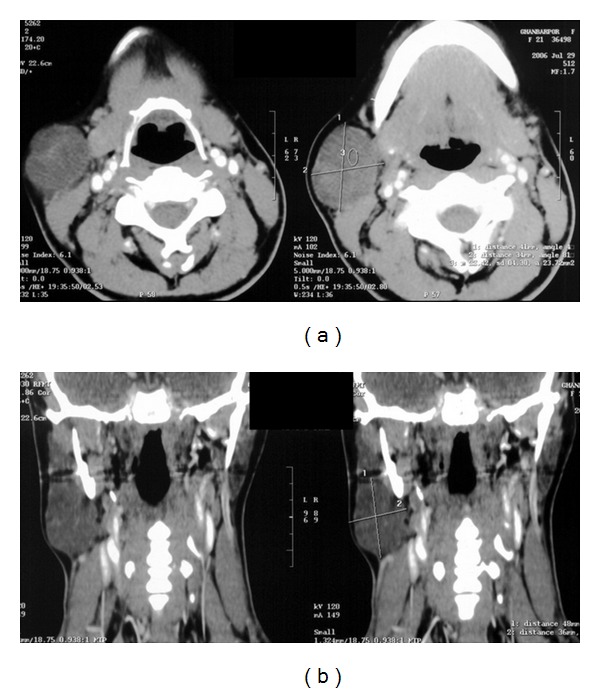
Contrast-enhanced CT scan in axial views of neck. A 36 × 48 mm soft tissue mass shown in the right side of the neck, in association of submandibular salivary gland and extra sheath of carotid, and without any invasion to peripheral tissues.

CT scan of the neck showed a 36 × 48 mm soft tissue mass on the right side of the neck, in association with extra sheath of carotid on the sternocleidomastoid muscle with medial displacement of carotid vessels (Figure 1). The mass showed a fine enhancement after contrast injection. CT scan was compatible with a lymphadenopathy.

The patient underwent total resection of the mass with diagnosis of primary cervical tumor. Intraoperatively, the tumor was completely placed in the cervical region far from the tail of parotid.

**Figure 2:**
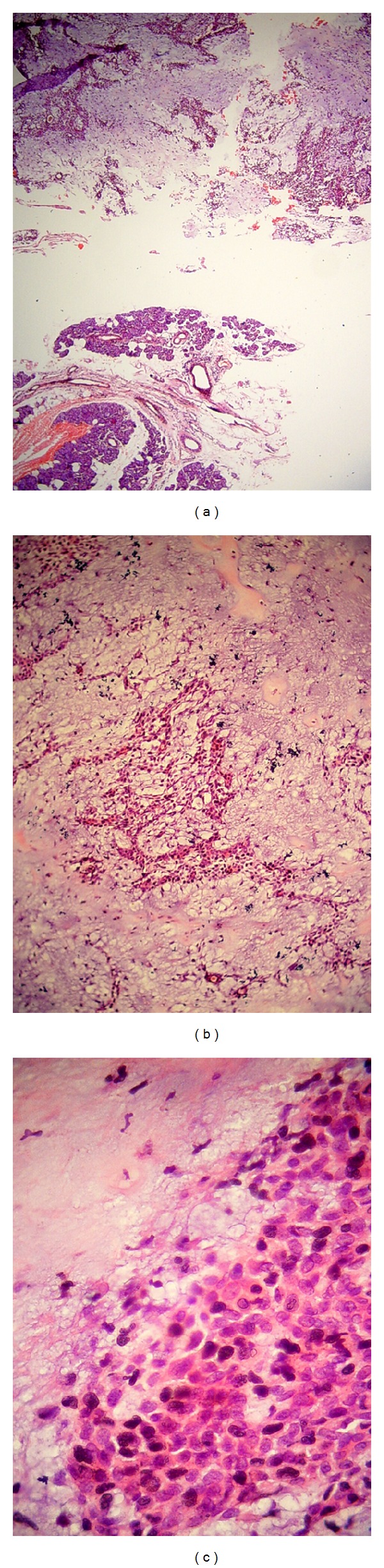
High power view of pleomorphic adenoma. The epithelial component is seen in the background of myxochondroid stroma.

Histopathological investigation confirmed the pleomorphic adenoma diagnosis (Figure 2).

The case report was approved by Ethics Committee of Tabriz University of Medical Sciences and the patient gave informed consent for this case presentation.

## 3. Discussion

Pleomorphic adenoma (benign mixed tumor) is the most common neoplasm of the salivary glands. They originate from an uncommitted reserve cell of the intercalated duct which has the potential to differentiate into epithelial and myoepithelial cells [2–5]. Pleomorphic adenoma of the minor salivary glands most commonly occurs in the palate [1–6].

Clinically, pleomorphic adenoma is characterized as a painless, slow-growing mass. Multiple primary pleomorphic adenomas are extremely rare. Pathologically, they are solitary, firm, round tumors. The cut surface is characteristically solid and can be hard, rubbery, or soft in consistent with whitish gray to pale yellow color. In contrast of major solitary glands, they are usually noncapsulated in minor solitary glands [7].

Common sites that minor salivary gland tumors arising are the palate, followed by the maxillary sinus, lip, cheek, tongue, and so forth [8].

Ishikawa et al. in Sao Paulo Medical University reported a 55-year-old man with nasal obstruction of many years ago that finally diagnosed as pleomorphic adenoma of maxillary sinus [9]. Khademi et al. in Shiraz Medical University of Iran reported a metastasizing mixed tumor of tongue base in a 19-year-old girl [10]. Takhur et al. reported a 35-year-old male with nasal obstruction, left aural fullness, and hyponasal speech because of nasopharyngeal pleomorphic adenoma [11]. Jin and Park, from Korea reported a 25-year-old woman that was referred for evaluating abnormal CXR that finally diagnosed as pulmonary mixed tumor [12].

During recent years, some other cases have been reported in rare regions such as oropharynx including tongue base [8], buccal space, pterygopalatine fossa, facial skin, trachea [1–13], breast [14], maxillary sinus [15], lateral wall of nasal, and nasal septum [16]. But present case is a new rare case involving neck.

## 4. Conclusion

 Pleomorphic adenoma may be rarely involving the neck. Although the prognosis is good, the choice treatment is the complete resection of the tumor. But, because of recurrence potential of this tumors, long-term followup is necessary.
